# Past, current, and future trends of red spiny lobster based on PCA with MaxEnt model in Galapagos Islands, Ecuador

**DOI:** 10.1002/ece3.3054

**Published:** 2017-05-28

**Authors:** Wladimir Moya, Gabriel Jacome, ChangKyoo Yoo

**Affiliations:** ^1^ Department of Environmental Sciences and EngineeringCollege of EngineeringCenter for Environmental StudiesKyung Hee UniversityYongin‐siGyeonggi‐doRepublic of Korea

**Keywords:** ArcGIS, Eco‐informatics, Galapagos Islands, MaxEnt, modeling, potential distribution, principal components analysis, predictor variable

## Abstract

In order to enhance in terms of accuracy and predict the modeling of the potential distribution of species, the integration of using principal components of environmental variables as input of maximum entropy (MaxEnt) has been proposed in this study. Principal components selected previously from the principal component analysis results performed in ArcGIS in the environmental variables was used as an input data of MaxEnt instead of raw data to model the potential distribution of red spiny lobster from the year 1997 to 2015 and for three different future scenarios 2020, 2050, and 2070. One set of six original environmental variables pertaining to the years 1997–2015 and one set of four variables for future scenarios were transformed independently into a single multiband raster in ArcGIS in order to select the variables whose eigenvalues explains more than 5% of the total variance with the purpose to use in the modeling prediction in MaxEnt. The years 1997 and 1998 were chosen to compare the accuracy of the model, showing better results using principal components instead of raw data in terms of area under the curve and partial receiver operating characteristic as well as better predictions of suitable areas. Using principal components as input of MaxEnt enhances the prediction of good habitat suitability for red spiny lobster; however, future scenarios suggest an adequate management by researches to elaborate appropriate guidelines for the conservation of the habitat for this valuable specie with face to the climate change.

1

**Research Highlights**

- Using principal components of PCA as input of MaxEnt instead of raw environmental data.
- Enhancing the accuracy and predictions of habitat suitability in red spiny lobster using MaxEnt.
- Analyzing the historical, current, and future trends of red spiny lobster in Galapagos under global warming.

## 2 INTRODUCTION

The Galapagos Islands are located in the equatorial eastern sector of the Pacific Ocean, approximately 1,000 km west of the coast of Ecuador, South America. Galapagos Islands consists of 234 islands, islets and rocks of volcanic origin, with a total land area of 7,985 km^2^, including 1,667 km of coastline (Szuwalski, Castrejon, Ovando, & Chasco, 2016).

During March 1998, the Galapagos Islands were included in a multiple‐use marine reserve nearly 138,000 km^2^ in size (Castrejon & Charles, 2014). Marine species are commercially harvested by local fishing communities, such as *Panulirus penicillatus*, red spiny lobster (RSL), which are the most economically valuable species in the Galapagos Marine Reserve (GMR) (Castrejon, 2011; Schiller, Alava, Grove, Reck, & Pauly, 2014).

The Intergovernmental Panel on Climate Change underpins the importance of conserving biodiversity in the face of climate change (IPCC, 2011). The relationship between species and its surrounding environmental variables have been of particular interest to ecologists (Kuemmerlen, Stoll, Sundermann, & Haase, 2016). Understanding these interactions are significant to predict current and future species distributions (Remya, Ramachandran, & Jayakumar, 2015). Species distribution modeling (SDM) is gradually used by scientists and policymakers as tools to investigate a wide variety of ecological problems. For the generation of SDM in Maximum entropy (MaxEnt), the environmental layers have been applied as hypothetical predictive variables using raw data, and associated to the geographical records of a particular specie show complications of spatial autocorrelation that can be defined as the degree of dependency of variables in geographical space (Anselin & Moreno, 2003). SDM obtained from a large dataset of associated environmental covariates often naturally result in multicollinearity, a statistical problem defined as a high degree of correlation among covariates as well in nonexperimental situations, where the researcher has no control of the risk associated to hypothetical factors related to independent variables.

To overcome this problem, principal components analysis (PCA) as a multivariate technique tool of ArcGIS can be apply as a predictor variable of environmental layers in order to incorporate as an input data of MaxEnt model instead of raw data of environmental variables. Many applications of PCA explain the reduction to a number of predictive variables that retain a high proportion of the original information (Tabachnick & Fidell, 2007).

Maximum entropy is considered to be the most consistent methodology in studies of distribution of species (Elith, Graham, Anderson, Dudik, & Ferrier, 2006; Hernandez, Graham, Master, & Albert, 2008; Phillips, Anderson, & Schapire, 2006; Wisz et al., 2008; Mateo, Croat, Felicisimo, & Muñoz, 2010; Aguirre‐Gutierrez et al., 2013) and has been described as especially efficient for handling complex interactions between response and predictor variables (Elith et al., 2011). In Galapagos Islands, MaxEnt model has not been used to predict the habitat suitability of RSL, nor focus on the impact of climate change on the distribution of the suitable habitat of this particular specie.

Therefore, the key contribution of the study is to retain the principal components (PCs) from PCA extracted from ArcGIS in environmental variables, whose eigenvalues explain more than 5% of the total variance and use them as input of MaxEnt instead of raw data, in order to determine how this approach can enhance the accuracy of the potential distribution of RSL considering 19‐year period and three future scenarios in GMR. Receiver operating characteristic (ROC) and the area under the ROC curve (AUC) as well Cohen's kappa statistic were used to validate the performance of the MaxEnt model.

## 3 METHODOLOGY

### 3.1 Study area

**Figure 1:**
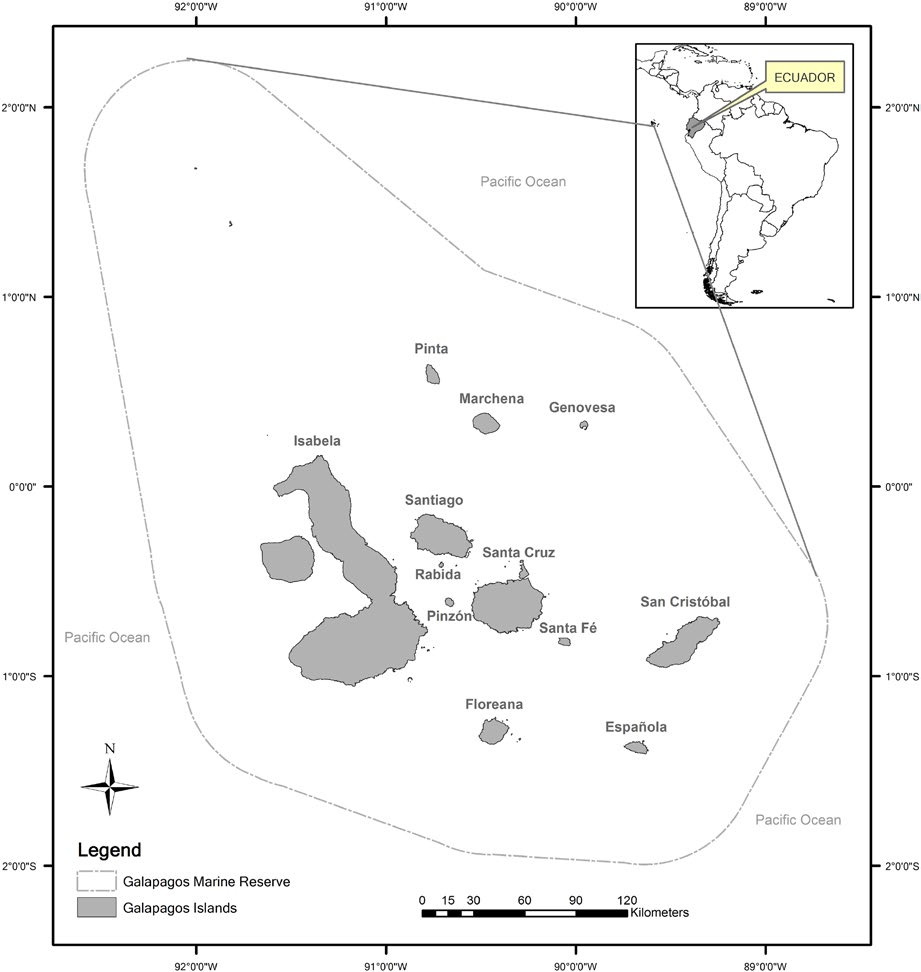
Study area for RSP in the Galapagos Islands—Ecuador

The study area considered for this study represents the Archipelago of Galapagos as presented in Figure 1. The total area covers 85,647 km^2^. The entire study area of GMR includes twelve islands that represent the summits of volcanoes that emerged from the sea approximately 1–3 million years ago. Three major ocean current influence the climate in the GMR: the Panama current, bringing warm water from the north; the Humboldt current, bringing cooler water from the south; and the upwelling subequatorial (or Cromwell) current, with highly productive cold waters (Hearn, 2008). The twelve islands within the study area are as follows: Isabela, Santa Cruz, Santa Fe, San Cristobal, Santiago, Floreana, Pinta, Pinzon, Genovesa, Marchena, Espanola, and Rabida.

### 3.2 Environmental predictors

For 19‐year period, six environmental variables were recorded as original climatic data: annual sea surface temperature (ASST); annual maximum air temperature (AMAT); annual mean air temperature (AMeAT); annual minimum air temperature (AMiAT); annual precipitation (AP); and annual relative humidity (ARH), taken from the data zone of the Charles Darwin National Foundation home page (http://www.darwinfoundation.org/datazone/climate/) from 1997 to 2015. Using PCA as a multivariate technique of ArcGIS, the eigenvalues of environmental variables that explained more than 5% of the total variance were selected as an input of MaxEnt.

Four environmental variables were considered for the future climate scenarios pertaining to the years 2020, 2050, and 2070 taken from Climate Change Scenarios, GIS program home page https://gisclimatechange.ucar.edu/gis-data. The environmental variables recorded were as follows: Total precipitation (PPT); surface temperature (TS); maximum air temperature (TASMAX); and minimum air temperature (TASMIN). The model simulation selected for futures scenarios was IPCC Climate Change Commitment Scenario (2000–2099). Using PCA technique in the environmental variables where its eigenvalues explained more than 5% was selected as input of MaxEnt. Table 1 shows all environmental variables considered in the study that had a spatial resolution of 1 km^2^.

**Table 1:** Original environmental variables used in the study

(a) Environmental variables in the 19‐year period (1997–2015)
AMAT = Annual maximum air temperature
AMeAT = Annual mean air temperature
AMiAT = Annual minimum air temperature
ARH = Annual relative humidity
ASST = Annual sea surface temperature
AP = Annual precipitation
(b) Environmental variables for future scenarios (2020, 2050 and 2070)
PPT = Total precipitation
TS = Surface temperature
TASMAX = Maximum air temperature
TASMIN = Minimum air temperature

### 3.3 Specie presence records

RSL (*Panulirus penicillatus)* is the specie most widely distributed of the spiny lobsters, ranging throughout the Indo‐Pacific, Red Sea, and eastern tropical Pacific Islands including the Archipelago of Galapagos, where it is found around most islands and islets, inhabiting the shallow rocky (Hickman & Zimmerman, 2000). This specie is gregarious and may often be found in groups of more than 20 individuals of different sizes, in submerged caves and lava tunnels (Hearn, 2008)

Presence data for “*P. pennicilatus*” were extracted from the History of Marine Animal Populations (HMAP)—GMR, Ecuador III (HMAP Data Pages), and previous monitorings performed by researches. A total of 39,150 presence records pertaining to the twelve islands for RSL were utilized to predict the potential distribution in the 19‐year period. The data were collected by interviewers and fishery observers from fishers at Puerto Ayora, Baquerizo Moreno, and Puerto Villamil on a daily basis during each fishing season, which usually lasted from September to December (with some exceptions).

Data provide the catch and effort statistics, the fishing method (Hookah diving), effective fishing hours (5 hr—average), number of divers (15 maximum), vessel type (fiberglass or wood), departure and landing port, and departure and arrival date. In addition, more than 150,000 measurements of total, carapace, and tail lengths of red lobsters were taken between 1997 and 2011. In most cases, these three types of lengths measurements were not registered for each individual.

For future climate scenarios pertaining to the years 2020, 2050, and 2070, the prediction of the occurrence of RSL was assessed using logistic regression model (LRM). The prediction of the occurrence of RSL for 2020 was performed through factor analysis (FA) as a first step, in which two independent factors were derived from the annual average from the years 1997 to 2015 considering the environmental variables selected previously from PCA. Thus, LRM was estimated and parametrized based on the two independent factors taken from the FA and the RSL presence in 2015 to predict the occurrence presence in 2020.

Similar procedure was conducted for the years 2050 and 2070, in which two independent factors were derived by FA. To predict the occurrence presence for RSL in 2050 and 2070, LRM was estimated and parametrized based on the two factors' scores of 2020 and 2050, and the predicted occurrence presence of RSL in 2020 and 2050, respectively.

### 3.4 Modeling procedure

ArcGIS 10.5 was used in order to reach the objective of this study. Six environmental variables recorded from 1997 to 2015 were imported to ArcGIS as an original raw data. The raw data were transformed to raster format and were analyzed annually starting from the year 1997. The environmental variables were cut within the study area. The “extract by mask” tool was used for each of the environmental variables in order to have the same extent and same cell of the study area. Using composite bands tool, ArcGIS creates a single raster dataset from multiple bands which means that all the six environmental variables were combined in a one multiband image in raster format. Nearest neighbor was used in the resample of the data during display. This multiband image was used as input of PCA to generate the respective predictor variables taking into account the same processing extent as the study area. The results of PCA were used as input of MaxEnt together with the presence records of RSL. The same procedure was conducted for the years 1998 to 2015 and for future scenarios (2020, 2050, and 2070). As a result, a range between two and four PCs among the six original environmental variables were selected by year to predict the potential distribution of RSL for the period 1997–2015, and four PCs for future scenarios, based on the percentage of variance explained by eigenvalues (>5%).

**Figure 2:**
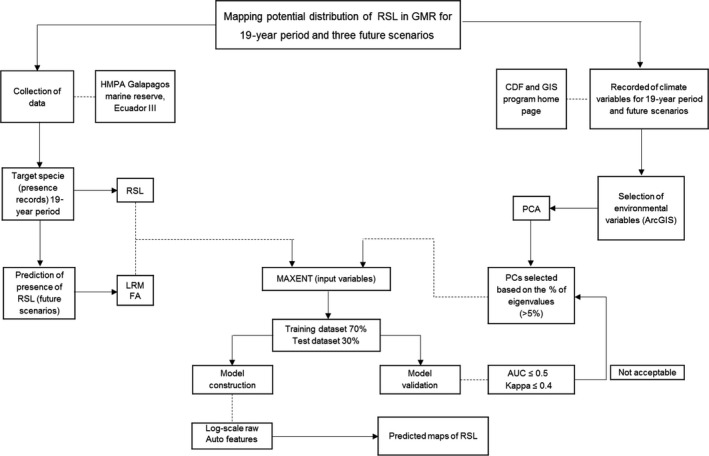
Generation of species distribution models using principal components as input of MaxEnt

The format supported by MaxEnt is ASCII (.asc); for this reason, each variable was reformatted to ASCII using the “Convert Raster to ASCII” tool. All predicted variables used in the model had 30 arc‐second spatial resolution (1‐km spatial resolution). Figure 2 illustrates the generation of species distribution models based on this approach.

Maximum entropy model performed best among many other species distribution models (Elith et al., 2006 and Ortega‐Huerta & Peterson, 2008). For ecological niche‐modeling using MaxEnt, the predicted variables obtained from PCA by each year must be loaded in ASCII format, and the presence occurrence data must contain the name of the species and geographical coordinates in CSV format generated in EXCEL software. For this purpose, ArcGIS 10.5 software was used to generate a shapefile of points using geographical coordinates to represent the presence of species, thereby identifying the points involved in developing the model. The definitive presence points were tabulated in EXCEL software. Each data point was placed in a single cell in the sheet, the name of the species followed by longitude and latitude geographical coordinates and separated by commas (,); in this way: “Panulirus_penicillatus, −90.04, −0.84,” this document was saved in CSV format (.csv).

The records of occurrence by each year as well the predicted occurrence for 2020, 2050, and 2070 and its coordinates were previously analyzed for consistency in order to eliminate pseudo‐absences generated outside the study area using the Spatial Analyst tool in ArcGIS software for environmental layers. The edition of environmental variables facilitated the transformation all environmental layers in order to obtain the same extent with the same pixel size (1 × 1 km) and the same position. Based on the spatial record occurrence of *Panulirus penicillatus,* the 70% of the records were used as a training model and the remaining 30% for validating the MaxEnt model. Runs were conducted with the default variable responses settings, and a logistic output format. Iterations were fixed as 5,000 and a convergence threshold as 0.00001. In order to avoid the overfitting of the test data, 0.1 was used as the regularization number (Phillips et al., 2006). The outputs generated by MaxEnt were transformed into raster format using the ArcMap tool in ArcGIS software for further analysis.

In order to have confidence in a predictive model, researchers such as Fielding and Bell (1997) and Farber and Kadmon (2003) described robust measures accepted as the best tools for evaluating model performance. AUC and Cohen's Kappa statistic were used in this study to assess MaxEnt model performance. An ROC test was applied for additional precision analysis. This measure estimates the relationship between AUC and the null expectation of bootstrap repetitions (Peterson, Papes, & Soberon, 2008). The evaluation of the model is based on forecast performance and includes the determination of a minimum threshold of the quantitative value produced for the potential presence of the communities. ROC figures and AUC values were obtained directly from the analysis of MaxEnt. Values vary between 0 and 1, where 1 indicates high performance, and values lower than 0.5 indicate low performance (Luoto, Poyry, Heikkinen, & Saarinen, 2005; Elith et al., 2006).

The data management tool was used to calculate the kappa statistic according to a classification map based on a set of polygons and random points created in the classification maps created in ArcGIS software. Zhang, Liu, Sun, and Wang (2015) suggested the following ranges of agreement of the kappa statistic (*K*): <0.4, poor; 0.4–0.8, useful; and > 0.8, good to excellent. Kappa statistic ranged from −1 to +1, where +1 indicates excellent agreement between predictions and observations and values of 0 or less indicate agreement no better than random classification (Zhang et al., 2015).

## 4 RESULTS

**Figure 3:**
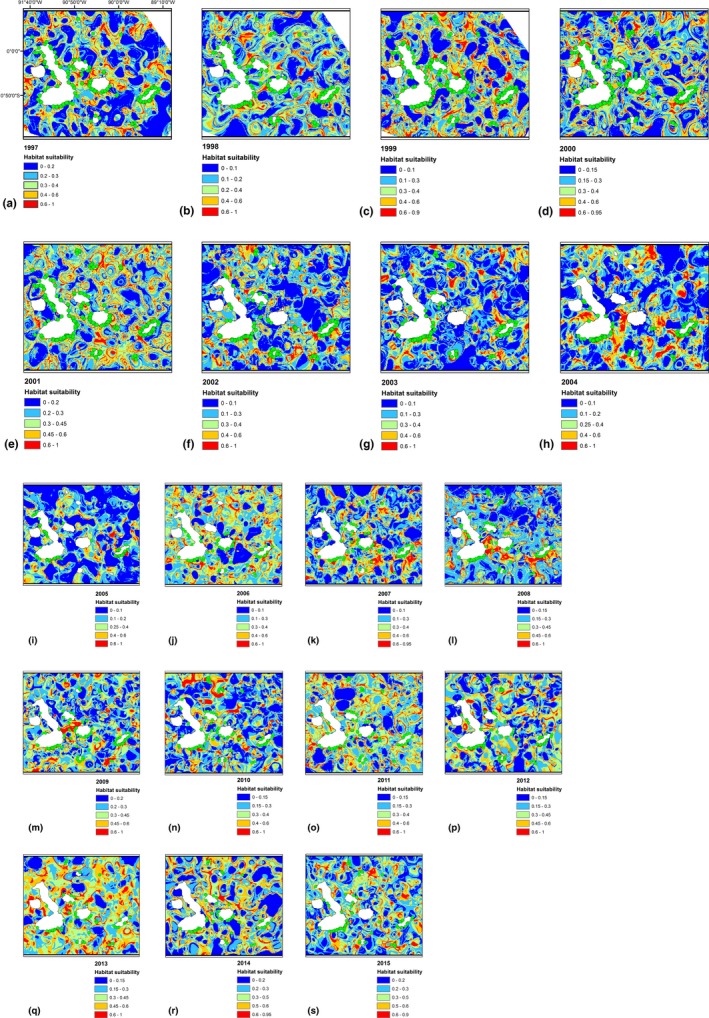
Distribution maps for red spiny lobster (RSL) in Galapagos marine reserve for the 19‐year period (a) 1997, (b) 1998, (c) 1999, (d) 2000, (e) 2001, (f) 2002, (g) 2003, (h) 2004, (i) 2005, (j) 2006, (k) 2007, (l) 2008, (m) 2009, (n) 2010, (o) 2011, (p) 2012, (q) 2013, (r) 2014, (s) 2015. Green dots represent the presence of the RSL, warmer colors indicate high probability of suitable conditions, and blue color indicates low predicted probability of suitable conditions

**Figure 4:**
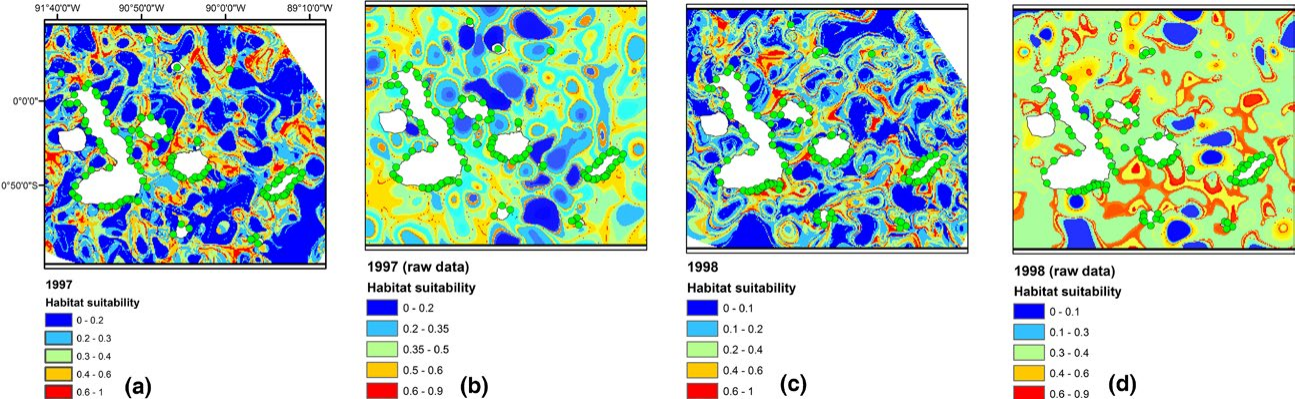
Distribution maps for red spiny lobster (RSL) in Galapagos marine reserve for the years 1997 using (a) principal components as input of MaxEnt and (b) raw data. Green dots represent the presence of the RSL, warmer colors indicate high probability of suitable conditions, and blue color indicate low predicted probability of suitable conditions

Table 2 summarizes the number of PCs predicted from PCA included in the model based on the percentage explained by eigenvalues (>5%) for the period 1997–2015 as well future scenarios (2020, 2050, and 2070). The analyses of PCA shows that in the most of the years, the variables such as AMAT, AMeAT, AP, and ARH were the variable predictors that contributed with the most variance among the original variables, explaining more than 95% of the total variance. Figure 3 shows the potential distribution of RSL along the 19‐year period, and Figure 4 shows the predicted potential distributions for futures scenarios pertaining to the years 2020, 2050, and 2070. Figure 3 illustrates the distribution of RSL across the GMR and its habitat suitability. The blue color shows unsuitable habitat for RSL, red color shows good suitable areas, and green dots show the presence occurrence of RSL. The unsuitable areas of RSL represented by blue color decreases from the years 1997 to 2001 in a range between 0 and 17%, by the other hand, the red zones which represents good suitable habitat, slightly increase in this period showing a range between 60 and 99% in terms of habitat suitability. The presence records of RSL in this period remain constant year by year. The most influence environmental variables during this period were AMAT, AMeAT, AMiAT, and SST. Between the years, 2001 and 2002, the areas with good habitat suitability increases (95%–98%), maintaining this tendency until 2005. The environmental predictors that influenced in the habitat suitability through this period were AP, ARH, ASST, and AMeAT. The presence records of RSL decreases in areas of good habitat suitability due to high precipitations occurring at this time. Changes between the years 2006 and 2007, in terms of habitat suitability, slightly decreases (97%–95%) maintaining the tendency until 2015 (93%). The most influence variables during this period were AP, ARH, ASST, and AMeAT.

**Table 2:** Number of principal components (PCs) extracted from principal components analysis during the 19‐year period and for future scenarios (2020, 2050, and 2070) based on percent explained by eigenvalues (>5%)

Year	Number of PCs	% of variance explained
1997	2	96.71
1998	2	95.26
1999	3	94.40
2000	4	98.03
2001	3	96.45
2002	2	98.34
2003	2	96.41
2004	2	92.71
2005	3	96.75
2006	4	95.77
2007	3	97.68
2008	2	97.13
2009	3	94.79
2010	3	98.10
2011	3	98.30
2012	3	96.66
2013	4	98.68
2014	3	95.84
2015	3	95.10
2020	4	99.97
2050	4	99.99
2070	4	99.94

The key point by applying this approach for this study is explained in Figure 4, where the year pertaining to 1997 and 1998 was taken because during this years, the phenomenon called El Nino was occurred in the equatorial zone affecting the Archipelago of Galapagos in terms of biodiversity. Figure 4a with 4b and 4c with 4d were compared in terms of prediction and habitat suitability, showing the distribution maps of RSL using PCs as input of MaxEnt and raw data, respectively. Using PCs in Figure 4a,c showed better predictions and better habitat suitability compared with raw data in Figure 4b,d. Good suitable areas represented by red color explained 98% and 99% in Figure 4a,c, respectively, in terms of prediction in which RSL can survive rather than raw data which explained 91% and 92% in Figure 4b,d, respectively. In terms of AUC values and Kappa statistic, the prediction using PCs was 0.92 and 0.98, in comparison with raw data that were 0.81 and 0.85 in the years 1997 and 1998, respectively.

Table 3 shows the contribution of each environmental variable for the period 1997–2015 as well for future scenarios (2020, 2050, 2070), being AMAT, AMeAT, AP, ASST, the environmental predictors with high contribution in the potential distribution of RSL in the 19‐year period and PPT, TS, TASMAX, and TASMIN for future scenarios. Table 4 shows the results of AUC and Kappa statistics from 1997 to 2015 and for futures scenarios 2020, 2050, and 2070. AUC values show good predictions for all the years (>0.85); however, Kappa values show better predictions (>0.95) than AUC values.

**Table 3:** Contribution of the variables during the 19‐year period and future scenarios in the modelling of the red spiny lobster distribution

Year	Variable contribution
1997	ARH (53.8%), AMAT (23%)
1998	AMAT (37%), ASST (33.5%)
1999	AMiAT (32.5%), AMAT (24.9%), ASST (22.8%)
2000	AMAT (30.4%), AMeAT (28.7%), ASST (26.7%), AMiAT (14.2%)
2001	ASST (34.6%), AMeAT (29.3%), AMiAT (18.2%)
2002	ARH (39.6%), AP (34.3%)
2003	AP (54.3%), ASST (14.6%)
2004	AP (35.6%), ARH (25.2%)
2005	AMeAT (34.2%), AP (27.8%), ARH (22.9%)
2006	ASST (48%), AP (32%), ARH (19.9%), AMAT (12.3%)
2007	AMeAT (32.8%), ASST (23.4%), ARH (22.3%)
2008	ARH (37.3%), AP (20.2%)
2009	AP (40.3%), AMeAT (25.2%), ASST (18.6%)
2010	AP (42.3%), AMeAT (28.9%), ARH (19.9%)
2011	AP (41.5%), ASST (31.5%), AMeAT (14.5%)
2012	ASST (53.8%), AP (17.8%), AMeAT (15.1%)
2013	ARH (41.9%), AP (41.8%), AMeAT (9.3%), ASST (7%)
2014	ASST (45.7%), AMeAT (40.8%), ARH (13.5%)
2015	ASST (39%), ARH (32.9%), AMeAT (28.1%)
2020	ASST (44.3%), AMiAT (25.8%), AMAT (16.3%), AP (13.6%)
2050	AMAT (34%), ASST (30.5%), AMiAT (28.5%), AP (7%)
2070	AMAT (38%), AMiAT (30.7%), ASST (21.5%), AP (9.7%)

See Nomenclature section for explanations.

**Table 4:** Area under the curve (AUC) and Kappa values for red spiny lobster generated by MaxEnt and ArcGIS

Year	PCs		Raw data	
	AUC	Kappa	AUC	Kappa
1997	0.92	0.98	0.81	0.85
1998	0.94	0.96	0.84	0.87
1999	0.93	0.95		
2000	0.94	0.94		
2001	0.93	0.97		
2002	0.94	0.92		
2003	0.95	0.95		
2004	0.95	0.98		
2005	0.93	0.94		
2006	0.93	0.98		
2007	0.94	0.95		
2008	0.92	0.98		
2009	0.85	0.99		
2010	0.90	0.99		
2011	0.89	0.98		
2012	0.86	0.97		
2013	0.81	0.95		
2014	0.87	0.93		
2015	0.85	0.74		
2020	0.98	0.98		
2050	0.98	0.99		
2070	0.97	0.98		

## 5 DISCUSSION

This study applied PCs extracted from ArcGIS in order to use as input of MaxEnt instead of raw data with the purpose of enhancing the potential and future predictions of RSL in GMR based on this approach. Common approach as using raw data to modelling potential present and future species distributions in ecological field do not always represent the uncertainty associated with the variable selection. Using RSL as a target specie, the uncertainties can produce effect in the outputs of SDM. Using raw data as an input of MaxEnt considering all the environmental variables rather than PCs extracted from ArcGIS increases the possibility of overfitting SDMs and may lead to the likelihood of a forecasted extinction under climate change. For RSL, the prediction of potential present and future distribution maps shows a variety of unsuitable and suitable habitats depending on the number of PCs chosen from PCA; thus, the selection of the variables is very important in RSL due to its biological characteristics and requirements.

Statistical methods of model selection by itself are not enough to reject or accept a model (Burnham & Anderson, 2002), as indices such AUC and Kappa; however, statistical models are a measure of internal model performance, not a measure of ecological validity. Therefore, the accuracy of the current and future distribution of RSL does not guarantee that the prediction will be accurate.

PCA has been widely used in various fields of investigation. These studies concern either environmental variation (Janzekovic & Novak, 2012), the investigated species, or communities characteristics. In aquatic habitat studies, it has been applied for evaluation of aquatic habitat suitability, their seasonal, and spatial variation (Ahmadi‐Nedushan et al., 2006). When using PCs as predictor variables, it is necessary to minimize the autocorrelation before the selection in order to avoid negative effects in the modelling analysis. The prediction maps using raw data generally present a reduced number of suitable areas for RSL and less accuracy in terms of AUC and kappa; this is due to that the environmental variables are correlated between each other. Using PCA, it reduces the autocorrelation, resulting in a linear combination of the original environmental variables reducing the number of PCs of the variables.

The results show that the potential distribution maps obtained from PCA can be used as a tool of better prediction in SDMs due to the reduction of autocorrelation of predicted variables and its suitable results. The statistical validations demonstrate good model performance using PCs that can be used as a tool in the prediction of the potential and future distribution of species.

With face to the climate change, El Niño/Southern Oscillation (ENSO) plays an important role regarding future climate scenarios under greenhouse warming. ENSO has been known as the most important year‐to‐year fluctuation on the climate system on the planet (McPhaden et al., 1999). During the 1982/83 and 1997/98, the equatorial region of Ecuador and northern Peru suffered catastrophic floods due to the extreme El Niño events causing surface warming anomalies. The frequency of ENSO extremes is expected to increase based on Cobb et al. study. The warming pattern gives rise to an increase in rainfall in the equatorial Pacific particularly in the eastern part of the basin. The accelerated warming in the equatorial zone leads to an increased frequency of equatorward shifts of the Intertropical Convergence Zone (ITCZ), which characterized an extreme El Niño, and to increase frequency of extreme swings of the South Pacific Convergence Zone (SPCG) toward the equator. Atmospheric convection tends to follow maximum sea surface temperatures (Wenju, Santoso, Wang, & Wu, 2015).

**Figure 5:**
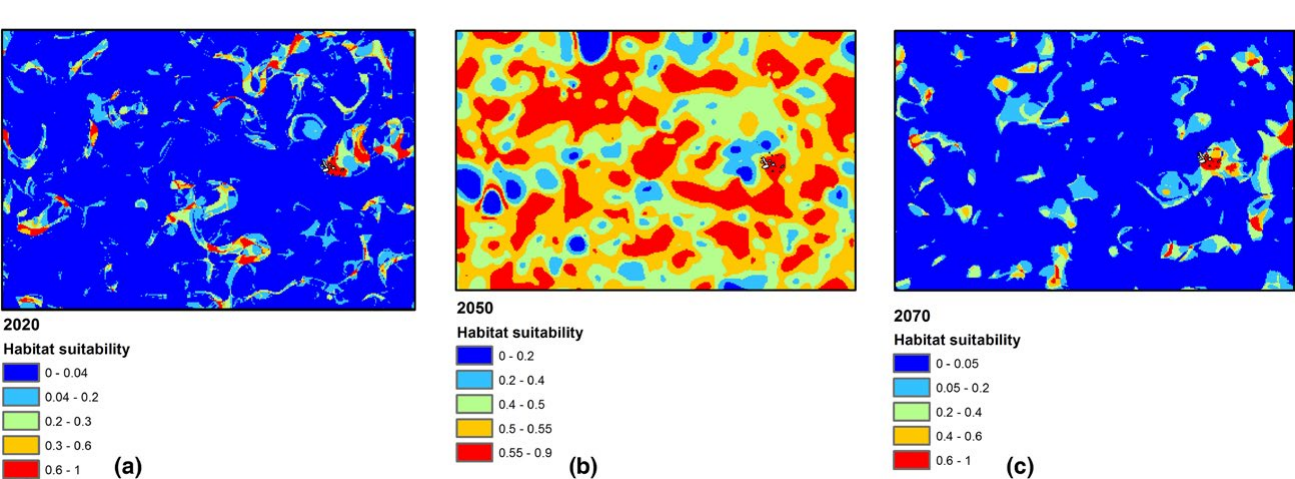
Distribution maps for red spiny lobster around Galapagos marine reserve for future scenarios pertaining to the years (a) 2020, (b) 2050, and (c) 2070. Warmer colors indicate high probability of suitable conditions and blue color indicates low predicted probability of suitable conditions

Galapagos is strongly influenced by ENSO. The ENSO causes extreme variability in the Galapagos every 2–7 years, with both warm (El Niño) and cold (La Niña) variations. Strong El Niño events cause increased sea surface temperature and air temperature above normal warm season conditions and are associated with increased rainfall. La Niña events caused below‐average air and sea temperatures, and decreased rainfall in the warm season (Trueman & d'Ozouville, 2010). The projected increase in extreme El Niño events more frequently creates favorable conditions for the occurrence of extreme La Niña events, expected to be double. The expected predictions of RSL for future scenarios regarding the relation to its environment and the influence of ENSO are shown in Figure 5. Figure 5a illustrates the case of 2020, in which areas of presence of RSL maintain similar trend with the last year analyzed in this study (2015) around GMR, and the environmental variables influenced in a positively way at this year. In 2050, the prediction of areas of good habitat suitability of RSL increases along the GMR and equatorial zone as shown in Figure 5b. This scenario can be explained due to the good environment conditions that can influence positively to RSL to increase its population. However, projecting to 2070, the environment conditions can be changed drastically due to the influence of its surrounding that can produce the decrease in suitable areas for the surviving of RSL. Considering these scenarios, the climate change has strong influence in the distribution of RSL; therefore, the suggestion for future studies is conducted an adequate management and elaborate an appropriate guidelines that support the decision‐making of the researches for the conservation of the habitat for this particular specie that is the most economically valuable in GMR in the face of global warming.

## 6 CONCLUSIONS

In this study, a PCA was applied as input of MaxEnt in order to model the potential distribution of RSL in GMR from the period 1997–2015 as well for future scenarios (2020, 2050 and 2070). The main conclusions are as follows:

1. The use of PCs of environmental variables as input of MaxEnt, instead of raw environmental data, can enhance the modelling of the potential distribution of a RSL in terms of accuracy and prediction in habitat suitability.
2. The environmental variables that had the most influence in the distribution of RSL from 1997 to 2015 were AMAT, AMeAT, AP, and ASST, and for future climate scenarios were PPT, TS, TASMAX, and TASMIN.
3. The Kappa index showed better prediction accuracy in comparison with AUC values, suggesting that it can be used as a more accurate tool for evaluating the quality and performance of species distribution models using MaxEnt.
4. Future research should focus on applying a continuous monitoring program in order to track biological and oceanographic changes in the marine ecosystem and conduct an adequate management for the conservation of the habitat of RSL in order to avoid its extinction with the face of global warming.

## CONFLICT OF INTEREST

None declared.

## NOMENCLATURE

**Table:** 

AUC	Area under the curve
AMAT	Annual maximum air temperature
AMeAT	Annual mean air temperature
AMiAT	Annual minimum air temperature
ARH	Annual relative humidity
AP	Annual precipitation
ASST	Annual sea surface temperature
FA	Factor analysis
GMR	Galapagos marine reserve
HMAP	History of Marine Animal Populations
IPCC	Climate Change Commitment Scenario
ITCZ	Intertropical convergence zone
LRM	Logistic regression model
PCA	Principal component analysis
PC	Principal component
PPT	Total precipitation
RSL	Red spiny lobster
ROC	Receiver operating characteristic
SDM	Species distribution modeling
TASMAX	Maximum air temperature
TASMIN	Minimum air temperature
TS	Surface temperature
